# Supplementary material for: From Science to Innovation in Aquatic Animal Nutrition: A Global TRL-Based Assessment of Insect-Derived Feed Technologies via Scientific Publications and Patents
Source: Animals (Basel). 2025 Oct 31;15(21):3174. doi: 10.3390/ani15213174 (PMC12607428; doi:10.3390/ani15213174)
Supplement: Supplementary file 1 [file animals-15-03174-s001.zip › animals-3919546-supplementary.pdf]

From Science to Innovation in Aquatic Animal Nutrition: A Global TRL-Based Assessment of Insect-Derived Feed Technologies via Scientific Publications and Patents

SUPPLEMENTARY MATERIAL

FIGURES

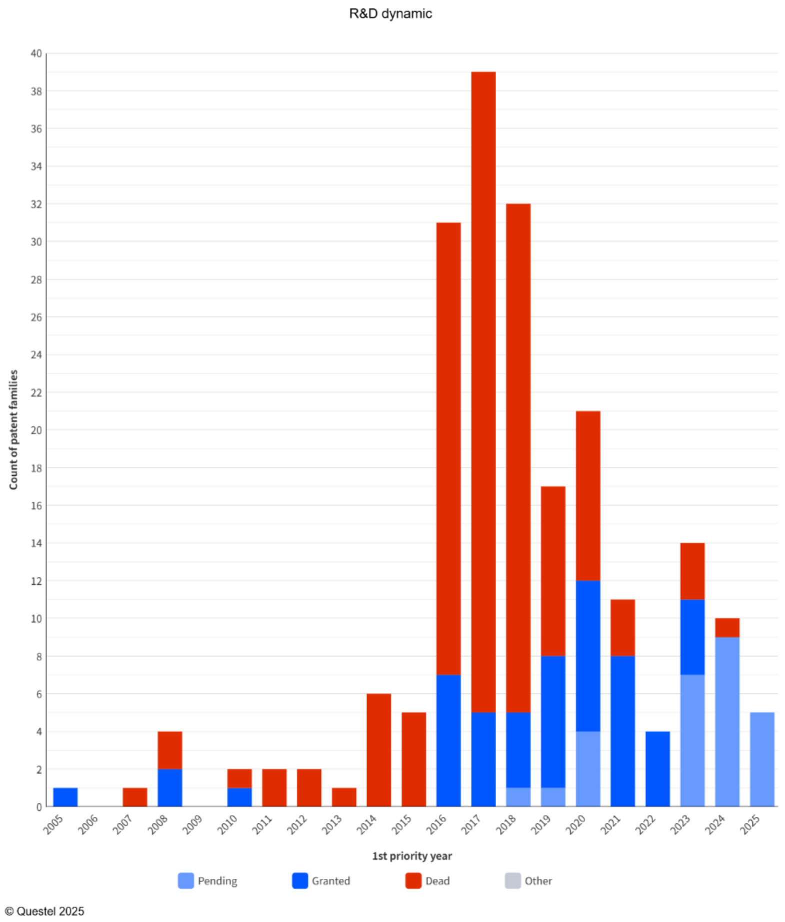

Figure S1. Patent families by first priority year, categorized by legal status: pending, granted, and dead (expired).

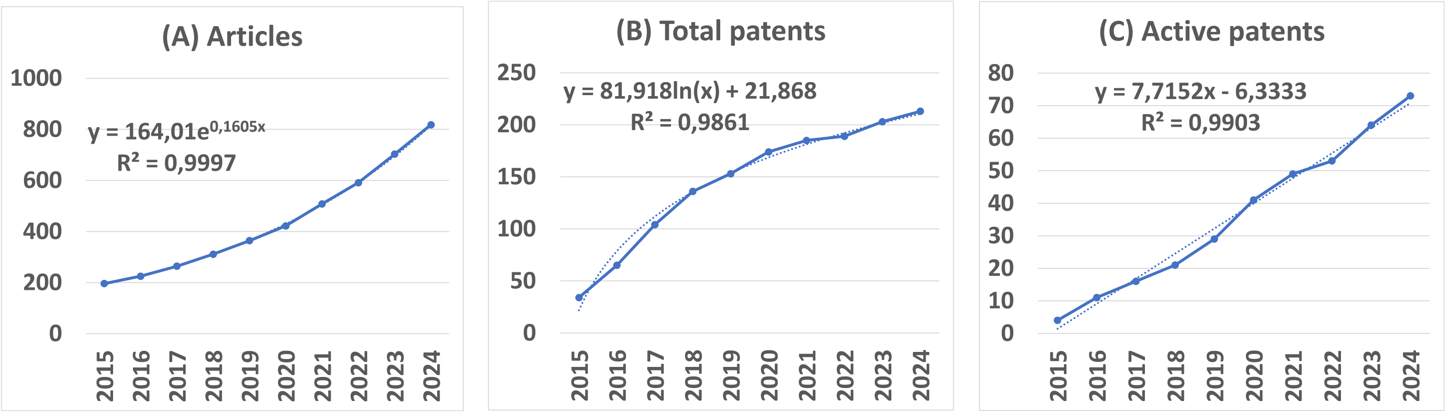

Figure S2. Fitted curves for time series used to analyze future trends: (A) Articles; (B) Total patents; (C) Active patents.

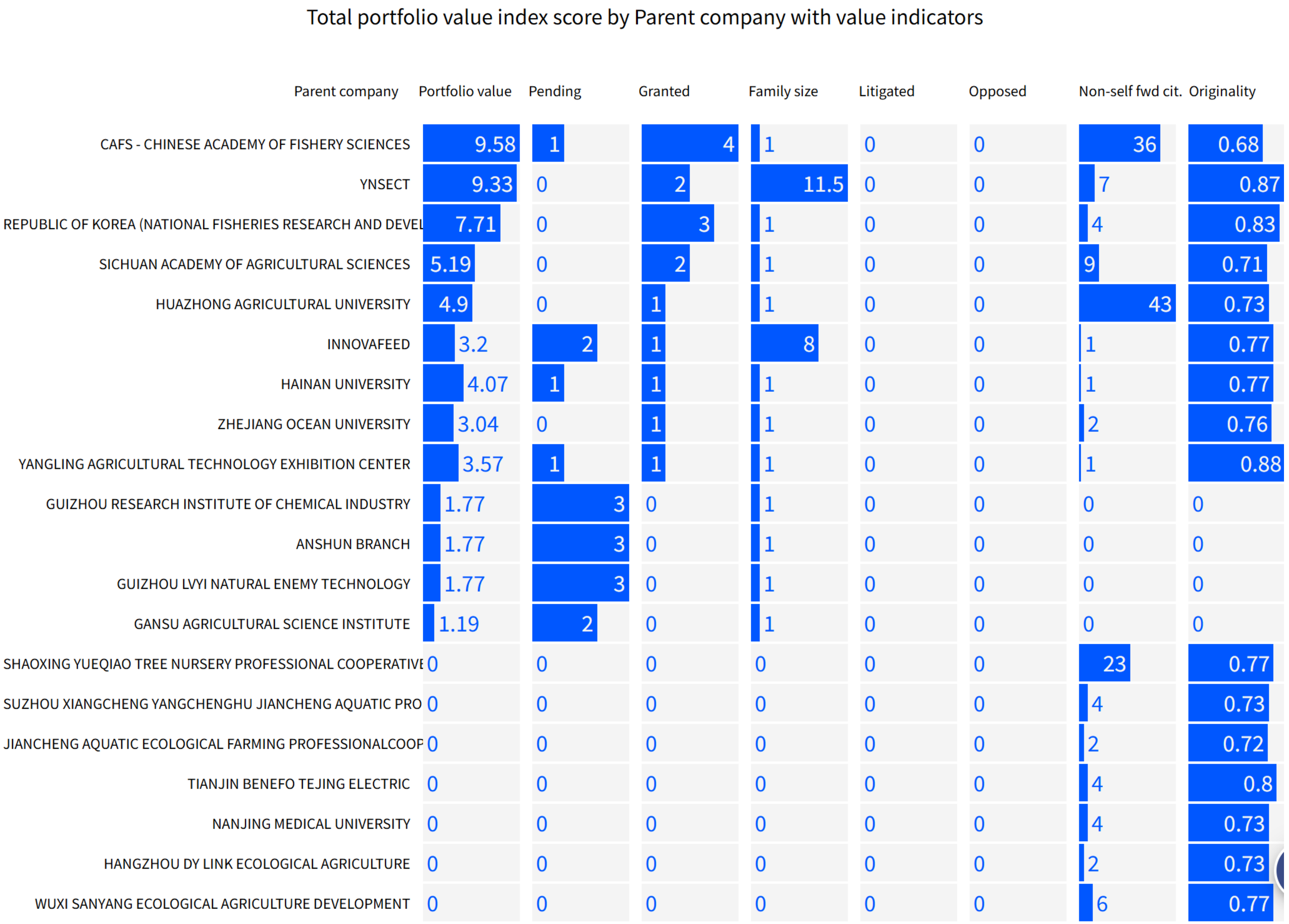

Figure S3. Impact indicators of the top 20 patent assignees, including portfolio value, pending patents, granted patents, family size, litigation, opposition, non-self forward citations, and originality.

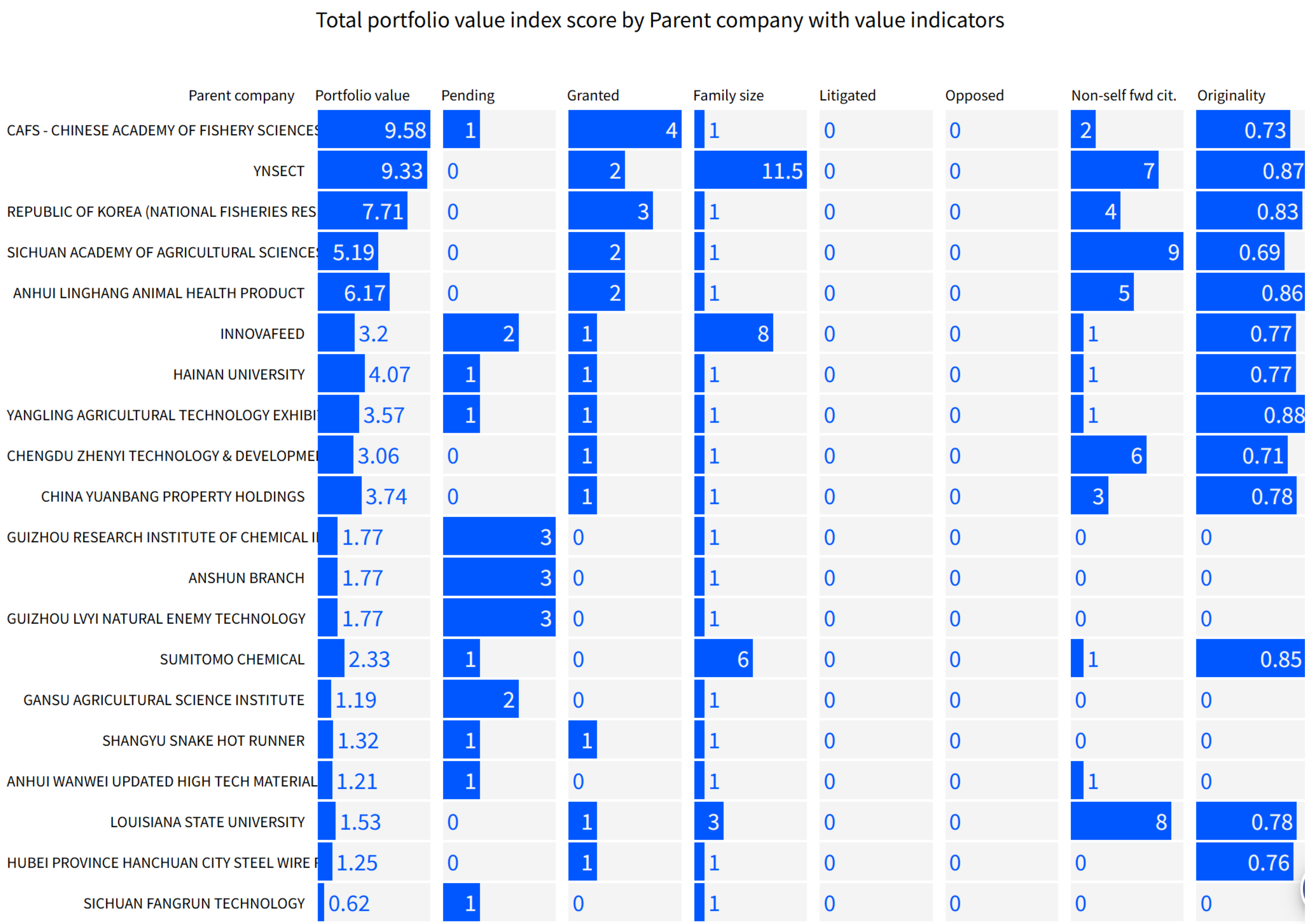

Figure S4. Impact indicators of the top 20 assignees of active patents (granted and pending), including portfolio value, pending patents, granted patents, family size, litigation, opposition, non-self forward citations, and originality.

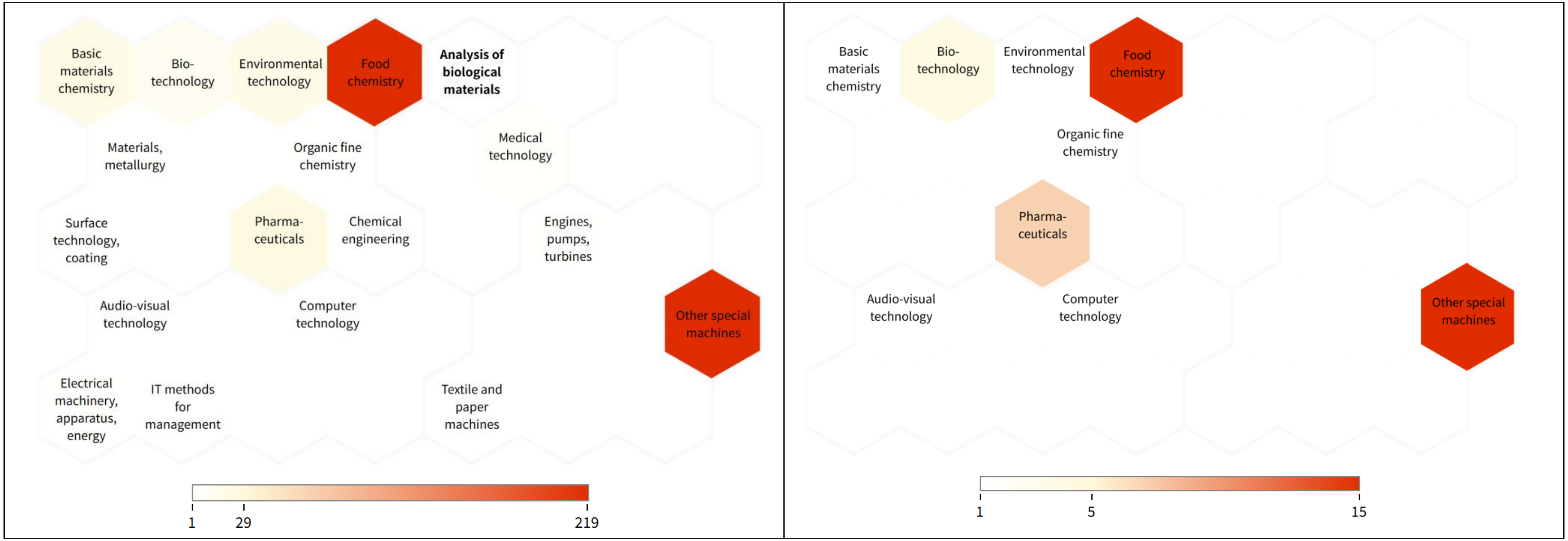

Figure S5. Technological domains of patents: (A) all patents; (B) active patents.

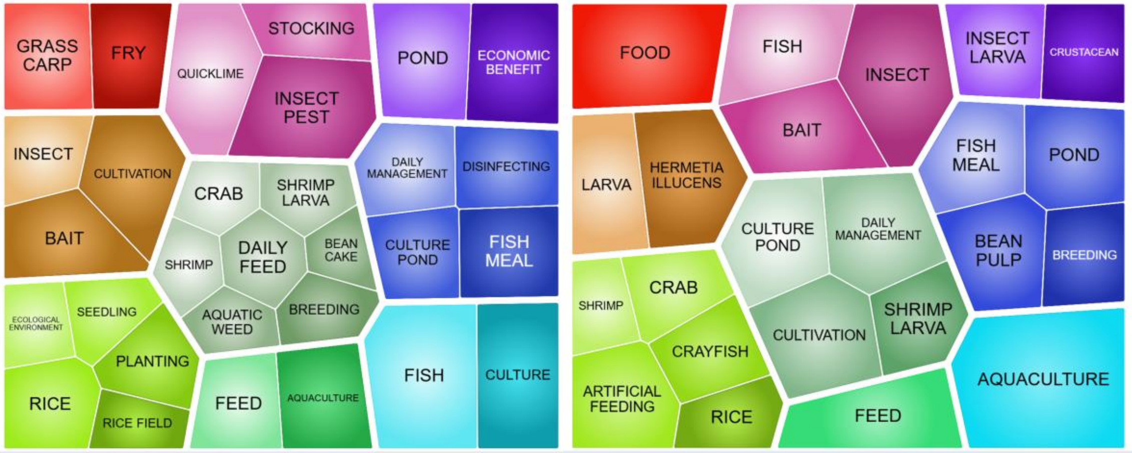

Figure S6. Concept clusters derived from patents: (A) all patents; (B) active patents.

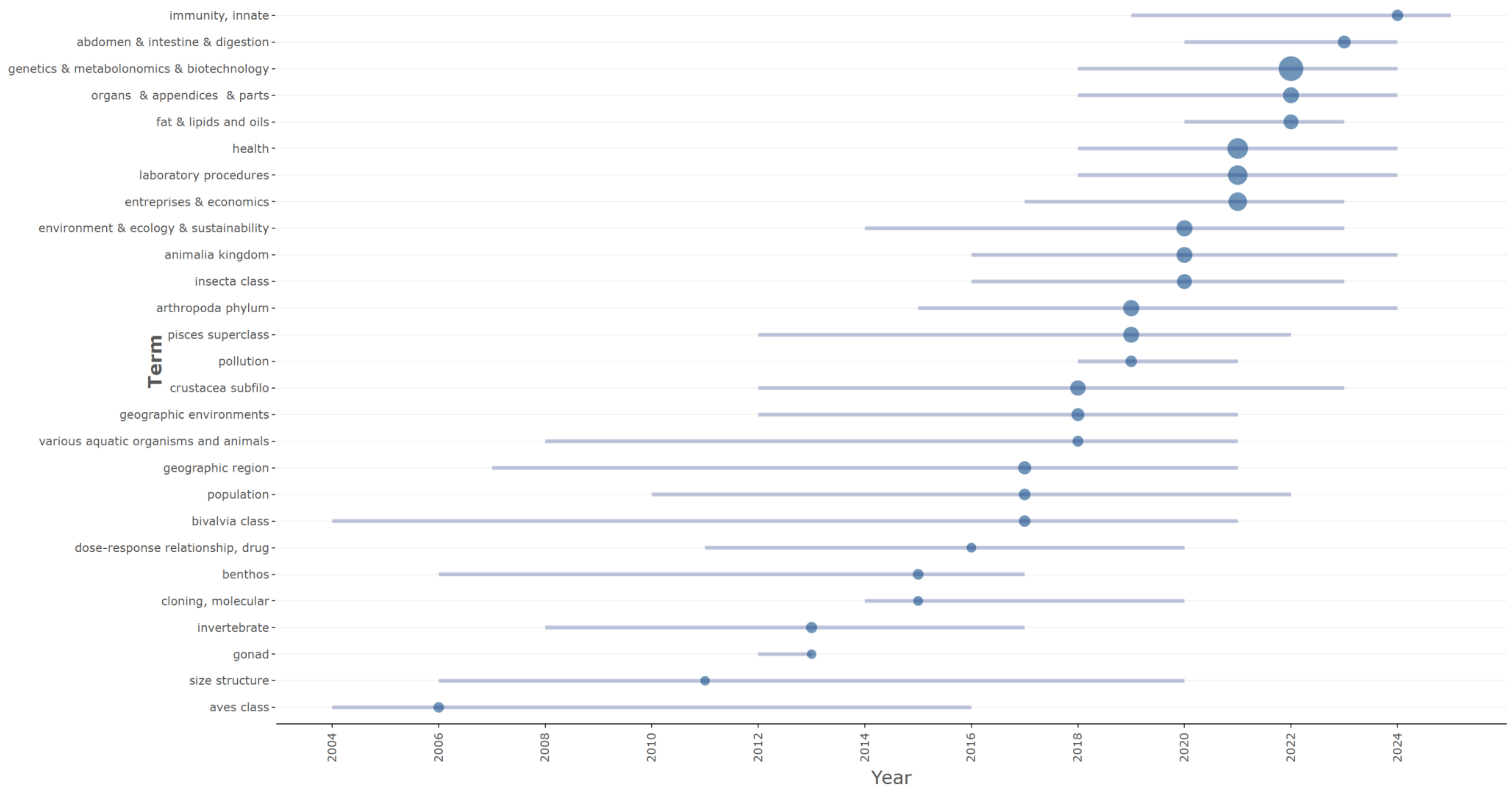

Figure S7. Annual evolution of trending topics/terms in the scientific articles.

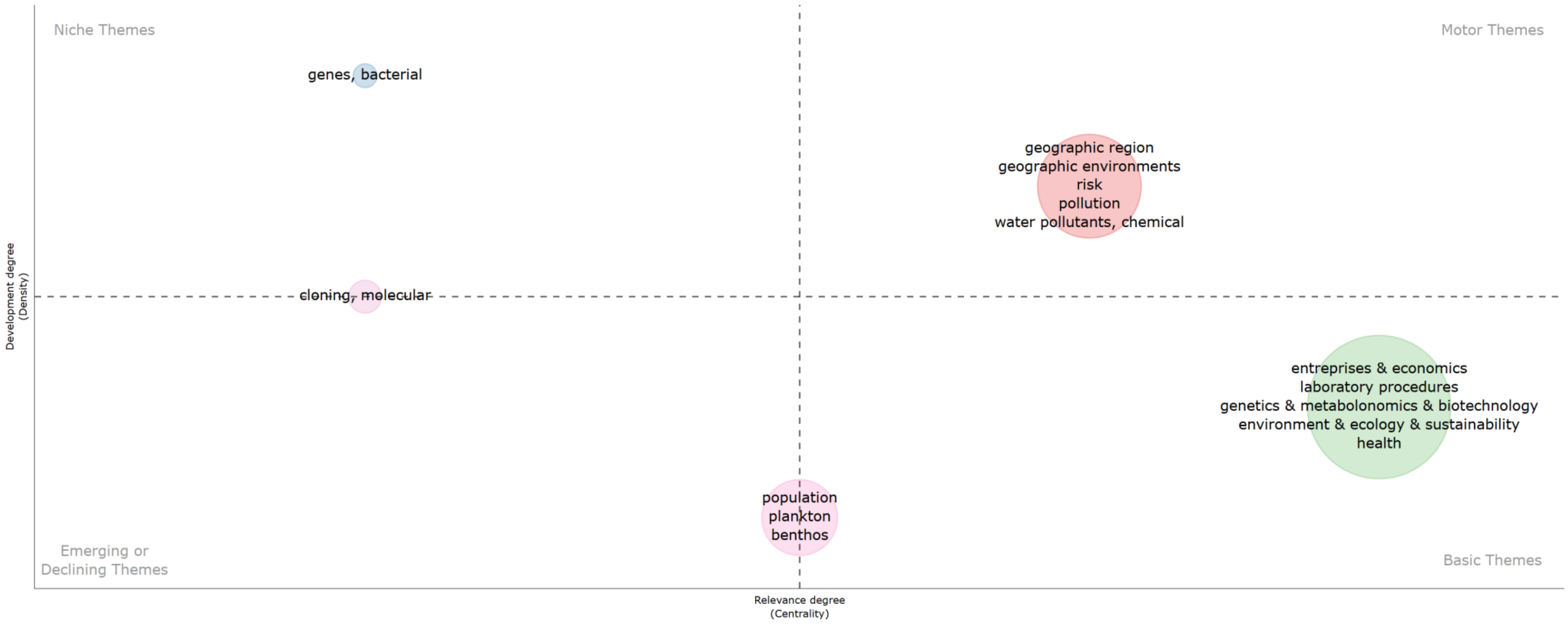

Figure S8. Thematic map of the articles, showing degree of development (density) versus degree of relevance (centrality), identifying niche themes, motor themes, basic themes, and emerging or declining themes.

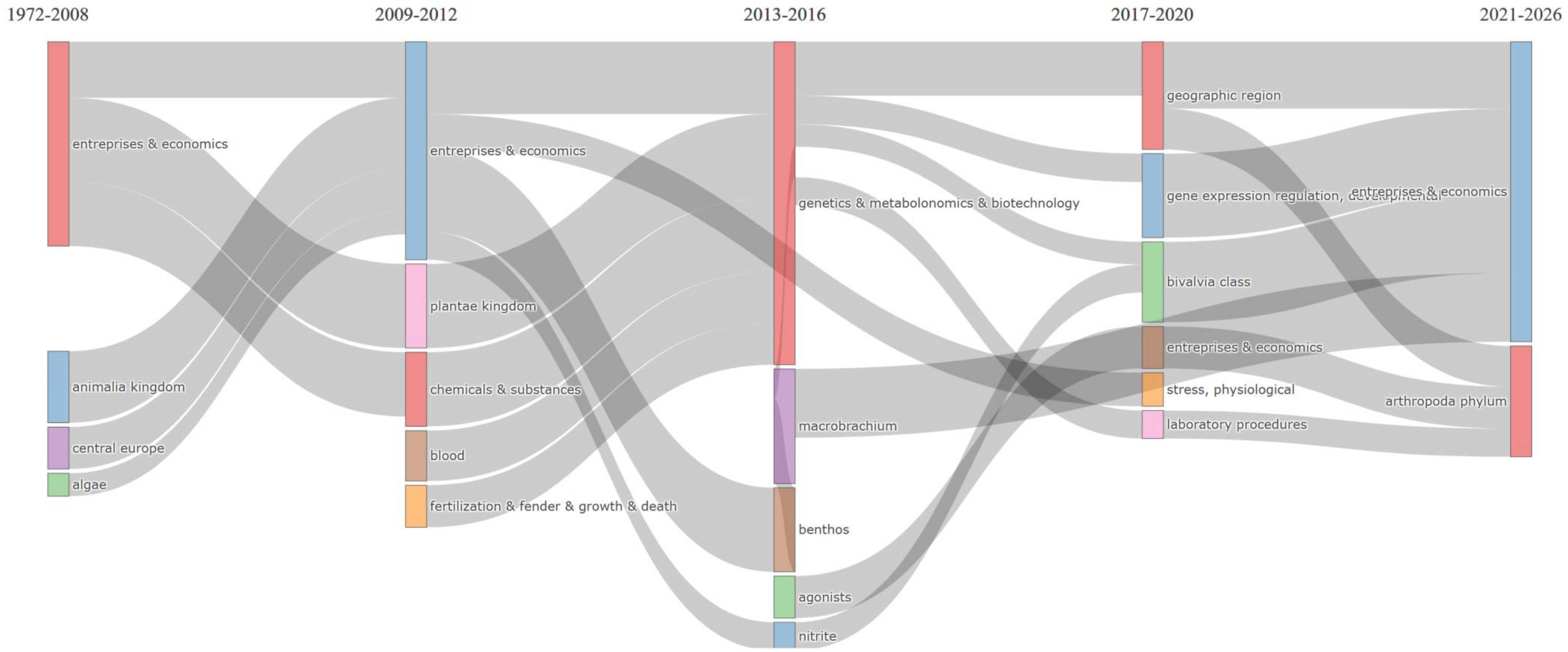

Figure S9. Thematic evolution of the articles across five time periods: 1972–2008, 2009–2012, 2013–2016, 2017–2020, and 2021–2025.

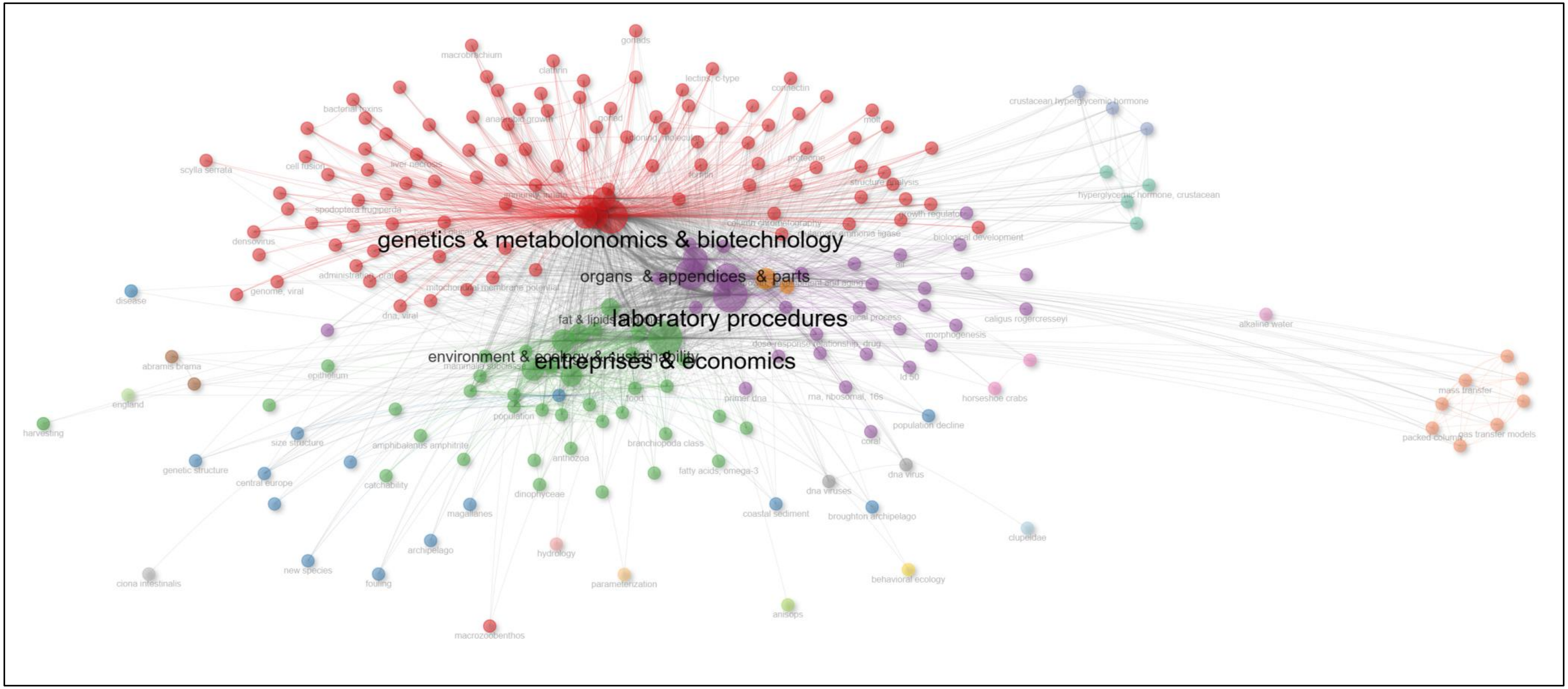

Figure S10. Thematic cluster map of the articles, identifying three main clusters: Cluster 1 (enterprises and economics), Cluster 2 (laboratory procedures), and Cluster 3 (genetics, metabolomics, biotechnology - reproduction, fertilization, gender, growth, and mortality).

From Science to Innovation in Aquatic Animal Nutrition: A Global TRL-Based Assessment of Insect-Derived Feed Technologies via Scientific Publications and Patents

SUPPLEMENTARY MATERIAL

TABLES

Table S1. Completeness of bibliographic metadata in the analyzed articles.

Metadata	Description	Missing Counts	Missing %	Status
AB	Abstract	0	0.00	Excellent
DT	Document Type	0	0.00	Excellent
LA	Language	0	0.00	Excellent
PY	Publication Year	0	0.00	Excellent
TI	Title	0	0.00	Excellent
TC	Total Citation	0	0.00	Excellent
C1	Affiliation	3	0.31	Good
AU	Author	3	0.31	Good
CR	Cited References	18	1.85	Good
DI	DOI	31	3.19	Good
SO	Journal	44	4.53	Good
DE	Keywords	88	9.05	Good
ID	Keywords Plus	340	34.98	Poor
RP	Corresponding Author	971	100.00	Completely missing
WC	Science Categories	971	100.00	Completely missing

Table S2(A). Published scientific articles by country (TRL3).

Country	Articles
China	213
United States	119
Italy	108
Norway	55
Spain	51
Australia	48
Germany	42
India	42
United Kingdom	41
Thailand	37
Brazil	35
Japan	31
Portugal	30
France	30
Malaysia	30
Canada	25
Egypt	25
Greece	24
Belgium	23
Czech Republic	23
Netherlands	22
Iran	20
Poland	19
Mexico	18
Chile	16
South Korea	16
Indonesia	14
Taiwan	12
Bangladesh	11
Turkey	11
Pakistan	10
South Africa	10
Undefined	10
Viet Nam	10
Ireland	9
Israel	9
New Zealand	9
Nigeria	9
Russian Federation	9
Kenya	8
Singapore	7
Sweden	7
Argentina	6
Denmark	6
Hungary	6
Philippines	6

Country	Articles
Switzerland	6
Austria	5
Finland	5
Laos	5
Saudi Arabia	5
Sri Lanka	5
Benin	4
Colombia	4
Croatia	4
Hong Kong	4
Malawi	4
Peru	4
Romania	4
Serbia	4
Uganda	4
Cameroon	3
Ecuador	2
Estonia	2
Ethiopia	2
Iceland	2
Monaco	2
Morocco	2
Slovakia	2
Ukraine	2
Afghanistan	1
Armenia	1
Azerbaijan	1
Bulgaria	1
Cyprus	1
French Polynesia	1
Ghana	1
Jordan	1
Kazakhstan	1
Latvia	1
Lithuania	1
Madagascar	1
Malta	1
Namibia	1
Nicaragua	1
Oman	1
Senegal	1
Solomon Islands	1
Tanzania	1
Uzbekistan	1
Venezuela	1
Zambia	1

Table S2(B). First-priority countries for all patent families (TRLs 4–5).

Country or Region	Patents
China	188
USA	8
South Korea	7
France	6
Japan	4
India	2
Australia	1
Cuba	1
Singapore	1
Taiwan	1
WIPO [1]	15
EPO [2]	2

[1] WIPO – World Intellectual Property Organization.
[2] EPO – European Patent Office.

Table S2(C). First-priority countries for active patents—those pending examination or granted (TRLs 4–5).

Country or Region	Patents
France	6
USA	5
China	1
Cuba	1
Japan	1
Singapore	1
Taiwan	1
WIPO [1]	
EPO [2]	

[1] WIPO – World Intellectual Property Organization.
[2] EPO – European Patent Office.

Table S2(D). Countries where patents have been filed beyond the first priority, indicating potential commercial markets (TRL 9).

Country or Region	Patents
China	63
India	10
South Korea	9
USA	9
France	6
Vietnam	6
Japan	5
Brazil	4
Australia	3
Ecuador	3
Indonesia	3
Malaysia	3
Peru	3
South Africa	3
Canada	2
Israel	2
Mexico	2
Singapore	2
Austria	1
Belgium	1
Bulgaria	1
Switzerland	1
Costa Rica	1

Country or Region	Patents
Germany	1
Denmark	1
Estonia	1
Spain	1
Finland	1
United Kingdom	1
Ireland	1
Italy	1
Lithuania	1
Luxembourg	1
Latvia	1
Malta	1
Netherlands	1
Philippines	1
Portugal	1
Sweden	1
Slovenia	1
Thailand	1
Taiwan	1
WIPO [1]	1
EPO [2]	5
APR [3]	1

[1] WIPO – World Intellectual Property Organization.

[2] EPO – European Patent Office.

[3] APR region - Asia and Pacific region.

Table S3. List of patents cited.

CN104285873	CN115669606	EP3678495
CN105994026	CN116548377	EP3863651
CN106035173	CN117502329	EP3911732
CN107114279	CN117981702	EP4120830
CN107306861	CN118542265	EP4166003
CN108496720	CN118556624	EP4464166
CN109156291	CN118844364	KR10-2287178
CN109418094	CN118923775	KR10-2287183
CN109673546	CN119631947	KR10-2373268
CN110537504	CN119924233	US10524490
CN110583903	CN120202975	US20200345039
CN112772470	CN120391359	US20210219570
CN112841088	CN221554394	WO2022/106790
CN114885870	EP1911764	WO2022/106791
CN115428755	EP2265132	WO2022/106792
CN115589972	EP2358193	
